# Supplementary material for: MetaRibo-Seq measures translation in microbiomes
Source: Nat Commun. 2020 Jun 29;11:3268. doi: 10.1038/s41467-020-17081-z (PMC7324362; doi:10.1038/s41467-020-17081-z)
Supplement: Supplementary file 10 — Supplementary Data 7 [file 41467_2020_17081_MOESM10_ESM.zip › File2/Confidence_VeryHigh_Taxonomy/3459_out.krona.html]

Javascript must be enabled to view this page.

members
magnitude
magnitudeUnassigned
count
unassigned
taxon
rank

3459\_out

14

2
14
superkingdom

phylum
7
1224

7
28211
class

356
7
order

69277
7
family

68287
7
genus

1670800
7
species

SRS015579\_contig\_number\_contig-100\_33729.33730SRS015960\_contig\_number\_7953SRS077849\_contig\_number\_contig-100\_32005.114807SRS097958\_contig\_number\_contig-100\_8224.46380SRS1055022\_contig\_number\_contig-100\_19953.57630SRS142503\_contig\_number\_contig-100\_7107.50657SRS144297\_contig\_number\_contig-100\_33708.33709

7
1239
phylum

class
7
186801

1
7
order
186802

SRS053356\_contig\_number\_42912

1898207
6
species

SRS013965\_contig\_number\_1647SRS015431\_contig\_number\_contig-100\_36032.171701SRS055017\_contig\_number\_19419SRS077194\_contig\_number\_4488SRS077392\_contig\_number\_37705SRS1041132\_contig\_number\_contig-100\_6005.6006
